# Mental Health Symptom Screening Among Adult Emergency Department Patients: A Cross-Sectional Study

**DOI:** 10.1016/j.acepjo.2025.100312

**Published:** 2026-01-13

**Authors:** Jacob Langkiet, Elizabeth Hutzel-Dunham, Jennifer L. Brown, Michael S. Lyons, Caroline Freiermuth, Jon E. Sprague, Joshua Lambert, Robert S. Braun, Andrew K. Littlefield, Jennifer A. Frey, Daniel Bachmann, Jason J. Bischof, Michael V. Pantalon, Rachel M. Ancona, Amanda Carroll, Dan Petrovitch, Katie P. Himes, Brittany E. Punches

**Affiliations:** 1 Department of Emergency Medicine, The Ohio State University College of Medicine, Columbus, Ohio, USA; 2 Department of Psychological Sciences, Purdue University, West Lafayette, Indiana, USA; 3 Department of Emergency Medicine, University of Cincinnati College of Medicine, Cincinnati, Ohio, USA; 4 The Ohio Attorney General’s Center for the Future of Forensic Science, Bowling Green State University, Bowling Green, Ohio, USA; 5 College of Nursing, University of Cincinnati, Cincinnati, Ohio, USA; 6 Department of Psychological Sciences, Texas Tech University, Lubbock, Texas, USA; 7 Long Island University, Brookville, New York, USA; 8 Department of Emergency Medicine, Washington University School of Medicine, St. Louis, Missouri, USA; 9 College of Nursing, The Ohio State University, Columbus, Ohio, USA

**Keywords:** emergency department, mental health screening, anxiety, depression, posttraumatic stress disorder

## Abstract

**Objectives:**

Mental health symptoms profoundly influence healthcare outcomes and utilization. Although mental health conditions are common in emergency department (ED) patients, routine, noncrisis screening is not consistently implemented; thus, problems may go unrecognized outside of psychiatric crisis presentations. We aimed to estimate the prevalence of current symptoms of anxiety, depression, and posttraumatic stress disorder (PTSD) among ED patients using validated screening tools.

**Methods:**

We conducted a secondary analysis of cross-sectional data from 1305 randomly selected, adult patients from 3 urban, academic EDs. Validated survey instruments included (i) Patient-Reported Outcomes Measurement Information System (PROMIS) Anxiety Short Form 8a, (ii) Emotional Distress Depression Short Form 8a, and (iii) Primary Care PTSD Short Form. The primary outcome was the proportion of ED patients screening positive for elevated symptoms. We also describe the demographic characteristics of the sample and select social determinants of health.

**Results:**

Participants had a median age of 48 years (IQR, 32-59); 53.1% were female and 49.0% were White. Socioeconomic challenges were common, with 57.2% unemployed and 45.5% reported annual income below $35,000. Overall, 706/1305 (54.1%, 95% CI, 51.4-56.8) screened positive for elevated symptoms for at least 1 mental health condition: anxiety 46.9% (*n* = 612; CI, 44.2-49.6), depression 36.8% (*n* = 480; CI, 34.2-39.4), and PTSD 21.9% (*n* = 286; 19.7-24.2). More than one-third (36.6%, *n* = 477) screened positive for 2 or more.

**Conclusion:**

Over half of ED patients screened positive for elevated symptoms. These findings underscore the epidemiologic burden of mental health symptoms in the ED and highlight the potential value of further research into brief, systematic screening during ED visits to identify and connect patients with appropriate mental health resources.

**The Bottom Line**

In this sample of noncrisis emergency department patients, over half screened positive for at least 1 mental health condition, and more than one-third screened positive for 2 or more. Anxiety was most common (46.9%). Younger age, female gender, and unemployment were associated with higher odds of a positive screen. These findings highlight the need for systematic mental health screening and targeted interventions in emergency care settings to address unmet behavioral health needs.

## 1 Introduction

### 1.1 Background

Mental health symptoms such as anxiety, depression, and posttraumatic stress disorder (PTSD) are common; yet, they are frequently missed in clinical documentation.^1^^,^^2^ Moreover, these symptoms significantly impact overall health and healthcare utilization.^3^^,^^4^ As of 2021, an estimated 57.8 million adults in the United States, approximately 1 in 5, were living with a mental health diagnosis.^5^ The global economic burden of mental health diagnoses reached $2.5 trillion in 2010 and is projected to reach $6 trillion per year by 2030.^6^^,^^7^ When symptoms are unrecognized or untreated, they create substantial challenges for both individual clinical care and healthcare resources.^1^

### 1.1 Importance

Emergency departments (EDs) increasingly serve as critical access points for individuals with unmet mental health needs, particularly those who may not engage with outpatient care. Although mental health symptoms are common among ED patients, systematic screening beyond crisis evaluation is not yet standard practice,^8^ and typical clinical pathways prioritize stabilization of acute medical complaints. Thus, noncrisis mental health conditions are often missed or deferred.^1^^,^^9^, ^10^, ^11^ Moreover, challenges such as psychiatric boarding and prolonged length of stay for patients with psychiatric diagnoses further strain overall ED resources and highlight the need for further characterization of the ED population.^12^ The percentage of ED visits related to mental health concerns increased dramatically from 11.5% in 2018 to 48% in 2021, reflecting both an increased need for and limited access to outpatient resources.^12^^,^^13^

### 1.3 Goals of This Investigation

Existing research has largely focused on crisis presentations or pediatric populations. Yet, adult ED visits for nonpsychiatric crises include a meaningful symptom burden. To complement the literature, we sought to quantify the prevalence of current, elevated symptoms of anxiety, depression, and PTSD among adults presenting to the ED for any reason using validated screening tools. Understanding the scope of mental health burden in the ED setting may inform future strategies for identification, intervention, and linkage to established care.

## 2 Methods

### 2.1 Study Design and Setting

This study is a secondary analysis of cross-sectional data collected as part of a parent study investigating pharmacogenetics of opioid use disorder (OUD).^14^ The enrollment was not restricted to patients with OUD; thus, we recruited adult ED patients regardless of OUD status or reason for visit. Data were collected across 2 academic health systems, which collectively comprise 3 urban EDs located in the state of Ohio. Two of these EDs are level 1 trauma centers, each accommodating over 70,000 visits annually. The third ED is an academic, level 1 trauma center, with approximately 40,000 visits per year. Each site’s Institutional Review Board approved all the documents related to the study protocol.

### 2.1 Selection of Participants

Between June 2020 and November 2021, adult ED patients who were not undergoing active resuscitation were identified for inclusion during their ED visit. The participant selection process began by randomizing all of the beds within each ED, after which research assistants (RAs) approached eligible individuals in each treatment area using a standardized, IRB-approved script. The script described the study purpose, described the types of questions involved, and disclosed compensation ($40 for survey and oral swab, $10 for optional blood draw). All patients present during the recruitment hours were considered. Non-English speakers, individuals under 18 years of age, in police custody with guard present, previously enrolled, physically restrained, or incapable of providing informed consent were excluded from the study. RAs obtained verbal consent and administered the survey during the ED visit. Participants could decline to participate at any point in the study.

### 2.3 Outcomes

The primary outcome measure was the proportion of participants screening positive for elevated symptoms of anxiety, depression, and/or PTSD.

### 2.4 Measurements

Trained clinical RAs administered a structured survey that included demographic information, select social determinants of health (SDOH), validated mental health screening tools including Patient-Reported Outcomes Measurement Information System (PROMIS) Anxiety Short Form 8a and Emotional Distress Depression Short Form 8a, and the Primary Care PTSD Short Form DSM-5.^15^^,^^16^ Each instrument assessed symptoms over the previous 7 days using validated wording (ie, “In the last 7 days…”). SDOH variables included education, employment, and income. No diagnostic assessments were performed.

The PROMIS Anxiety Short Form 8a and Emotional Distress Depression Short Form 8a each consist of 8 items that are rated on a 5-point Likert-type scale. The raw scores were converted to standardized T scores using PROMIS scoring manual procedures. Higher T scores indicate greater symptom severity. Cutoff scores for elevated symptoms followed published scoring guidelines.^16^ Participants were considered to screen positive for anxiety or depression symptoms if their T score fell within the mild, moderate, or severe range.

PTSD symptoms were assessed using the PC-PTSD-5, a 5-item binary response measure.^16^ Participants were initially screened for a lifetime traumatic event exposure and then subsequently administered the PC-PTSD-5 tool. A raw score greater than or equal to 3 was used to define a positive screen.^16^

### 2.5 Analysis

Descriptive statistics were used to summarize participant characteristics and prevalence of elevated symptoms (Table 1). Rates of the primary outcome are reported as proportions with 95% CI using Clopper-Pearson exact methods. T score distributions were described with means, standard deviations, IQR, and minimum and maximum values. Symptom severity categories were reported with counts and percentages. We explored associations between mental health symptoms and demographic variables (age, race, sex, education, income, and employment) using multivariable logistic regression models, reporting adjusted odds ratios for associations, Wald 95% CI, and *P* values. Analyses were conducted using IBM SPSS Statistics, version 29.0.1.1. Sensitivity analyses were conducted to assess the impact of missing data.

**Table 1:** Characteristics of study population screening positive for symptoms of posttraumatic stress disaster (PTSD), anxiety, and depression.

	Full sample *n* = 1305, %		Anxiety *n* = 612 (46.9%), %		Depression *n* = 480 (36.8%), %		PTSD *n* = 286 (22.0%), %	
Age–years, median (IQR)	48 (32-59)		44 (31-56)		44 (32-57)		48 (30-53)	
Race								
Black/African American	602	46.1	261	42.6	214	44.6	120	42.0
White	639	49.0	316	51.6	241	50.2	148	51.7
Other	64	4.9	35	5.7	25	5.2	18	6.3
Ethnicity								
Hispanic/Latino	37	2.8	19	3.1	16	3.3	9	3.1
Non-Hispanic/Latino	1268	97.2	593	96.9	464	96.7	277	96.9
Gender								
Male	604	46.3	242	39.5	202	42.1	105	36.7
Female	693	53.1	363	59.3	273	56.9	177	61.9
Education								
No high school diploma	201	15.4	93	15.2	83	17.3	47	16.4
High school diploma/equal	431	33.0	202	33.0	161	33.5	27	9.4
some college	384	29.4	192	31.4	147	30.6	69	24.1
College graduate	283	21.7	122	19.9	86	17.9	99	34.6
Employment status								
Employed	549	42.1	241	39.4	168	35.0	102	35.7
Unemployed	746	57.2	371	60.6	312	65.0	183	64.0

## 3 Results

### 3.1 Characteristics of Study Subjects

Of the 6176 patients screened, 3541 were potentially eligible and approached for enrollment. A total of 1305 participants (36.9%) consented and completed the survey. The median age was 48 years (IQR, 32-59), 53.1% were female, and 49.0% were White. Regarding SDOH, 57.2% were unemployed, 45.5% reported an annual income below $35,000, and 15.4% lacked a high school degree.

### 3.2 Main Results

Analysis identified that 54.1% (*n* = 706; 95% CI, 67.7-72.6) screened positive for elevated symptoms consistent with at least one mental health condition. Specifically, 46.9% (*n* = 612; 95% CI, 44.2-49.6) screened positive for anxiety, 36.8% (*n* = 480; 95% CI, 34.2-39.4) for depression, and 21.9% (95% CI, 19.7-24.2) for PTSD⁠. Additionally, 477 participants (36.6%) screened positive for 2 or more of these conditions.

PROMIS T scores were analyzed for insight into the range and severity of anxiety and depression. For anxiety, the mean T score was 54.6 (SD = 12.2) with scores ranging from 37.1 to 83.1 (IQR = 16.6). Of the participants, 14.4% were classified with mild symptoms of anxiety, 21.7% with moderate, and 10.8% with severe. Notably, 32.5% of all patients screened had moderate-to-severe anxiety symptoms. For depression, the mean T score was 51.2 (SD = 11.6), with a range of 38.2 to 81.3 (IQR = 21.2). Here, 14.4% of participants had mild symptoms of depression, 16.9% moderate, and 5.4% severe, with 22.3% of participants displaying moderate-to-severe depression symptoms. PTSD scores ranged from 0 to 5 (mean = 1.08, SD = 1.67). We had a small proportion of participants who did not complete all mental health symptom measures, 7.3% (*n* = 95) of participants had missing data for at least 1 screening instrument (anxiety, depression, or PTSD).

Statistically significant associations were found between elevated mental health symptoms and several demographic and SDOH variables (Table 2). Younger age was associated with elevated symptoms for all 3 conditions (anxiety: AOR, 0.98 95% CI [0.97, 0.98]; depression: AOR, 0.98, 95% CI [0.97, 0.99]; PTSD: AOR, 0.97, 95% CI [0.96, 0.98], and all *P <* .001). Gender and employment status were also key predictors. Female gender was associated with higher odds of elevated anxiety symptoms (OR = 1.56, 95% CI [1.23, 1.97], *P* < .001) and PTSD (OR = 1.51, 95% CI [1.14, 1.99], *P* = .004). Unemployment was strongly associated with elevated symptoms across all 3 domains: anxiety (AOR, 1.61; 95% CI [1.24-2.08], *P* < .001), depression (AOR, 1.84; 95% CI [1.41-2.40], *P* < .001), and PTSD (AOR, 1.84; 95% CI [1.35-2.5], *P* < .001). Regarding race, Black/African American patients had lower odds of screening positive for anxiety compared with White patients (AOR, 0.70; 95% CI [0.55-0.90], *P* = .005), but no significant differences were observed for depression or PTSD. In the adjusted models, educational attainment was not statistically associated with screening positive for mental health symptoms (all *P* > .05).

**Table 2:** Associations between demographic factors and screening positive for anxiety, depression, and PTSD.

	Anxiety			Depression			PTSD		
	AOR	95% CI	*P* value	AOR	95% CI	*P* value	AOR	95% CI	*P* value
Age (per year inc)	0.98	0.97-0.98	<.001	0.98	0.97-0.99	<.001	0.97	0.96-0.98	<.001
Gender (Ref: male)									
Female	1.56	1.23-1.97	<.001	1.22	0.96-1.55	0.11	1.51	1.14-1.99	0.004
Race (Ref: White)									
Black/African American	0.70	0.55-0.90	0.005	0.84	0.66-1.09	0.19	0.77	0.57-1.03	0.08
Other^a^	1.17	0.65-2.10	0.61	0.99	0.55-1.78	0.96	1.22	0.64-2.32	0.56
Ethnicity (Ref: non-Hispanic/Latino)									
Hispanic/Latino	0.72	0.33-1.55	0.40	0.95	0.44-2.05	0.90	0.66	0.27-1.63	0.37
Education (Ref: college grad or higher)									
No high school diploma	1.06	0.71-1.60	0.77	1.40	0.92-2.12	0.12	1.30	0.80-2.12	0.29
High school diploma/GED	1.07	0.76-1.50	0.72	1.18	0.83-1.69	0.35	1.27	0.84-1.93	0.26
Some college	1.23	0.88-1.73	0.23	1.27	0.90-1.81	0.18	1.32	0.88-1.99	0.19
Employment status (Ref: employed)									
Unemployed	1.61	1.24-2.08	<.001	1.84	1.41-2.40	<.001	1.84	1.35-2.50	<.001

OR, odds ratio; CI, confidence interval; Ref, reference group.

Values are from 3 separate multivariable logistic regression models for each outcome (Anxiety, Depression, PTSD).

Bolded results indicate statistical significance (*P* < .05).

95% CI is presented as [lower limit] – [upper limit].

*P* values are rounded to 3 decimal places; if *P* < .001, displayed as “<.001.”

a Other includes participants identifying as Asian, American Indian/Alaska Native, Native Hawaiian/Pacific Islander, other, and Biracial/Multiracial.

## 4 Limitations

Although a strength lies in the randomized sampling approach for enrolling patients from the emergency department, the identification of mental illness symptoms relied solely on self-reported data collected through interviews guided by a research assistant. This methodology introduces the possibility of recall bias or other forms of response bias.^17^ As no data were collected on nonrespondents, we were unable to compare these group differences. Additionally, the comprehensive survey instrument may have created a response burden on participants, potentially influencing data quality and completeness.

A small proportion of participants did not complete all of the measures related to mental health symptoms. We conducted a sensitivity analysis to assess whether missingness was associated with demographic characteristics and found no significant associations. However, we acknowledge the possibility of missing data, especially due to the sensitive nature of mental health symptoms. Thus, our reported prevalence is likely an underestimate of the burden of elevated mental health symptoms and our results should be interpreted accordingly.

This study should be interpreted with the environment of the ED in mind. Although these instruments are anchored to the past 7 days, they may incorporate both ongoing symptoms as well as the situational distress of the ED encounter. Additionally, the established cutoffs were validated in the general population, not in ED settings. This potentially may limit the interpretation of symptom severity. Future research should consider the development or validation of ED-specific norms for mental health symptom screening measures.

Finally, the study’s external validity may be limited by the enrollment rate, as 36.9% of eligible patients participated. Selection bias is possible if those who chose to participate differed systematically from those who declined. Chief complaint and prior psychiatric diagnoses were not systematically collected, limiting our ability to assess the representativeness of the sample. Furthermore, the study was conducted at urban academic medical centers, which may limit generalizability to other emergency care settings.

## 5 Discussion

In this cross-sectional analysis of adult ED patients, over half (54.1%) screened positive for at least 1 elevated symptom suggestive of a mental health condition, substantially exceeding national estimates.^2^ Notably, more than one-third (36.6%) screened positive for 2 or more conditions. Although screening positive using validated instruments does not constitute a formal diagnosis, identifying persons at increased risk is essential for early intervention and to determine who may benefit from additional assessment and referral. Our approach highlights unmet need for earlier identification of mental health concerns within ED care.

Our primary outcomes reflects any positive screen, including persons screening in the “mild” category. However, 32.5% of all patients screened had moderate-to-severe anxiety symptoms and 22.3% of participants displayed moderate-to-severe depression symptoms. Although clinical pathways for mild symptoms differ for those with moderate-to-severe symptoms, we believe identifying the full scope of symptom burden is a strength. The ED is a critical access point for persons often not accessing healthcare otherwise. This provides an opportunity to intervene earlier and potentially linkage to resources to promote long-term mental health wellness.

Our findings are consistent with prior research indicating that EDs serve as critical access points for individuals with unmet mental health needs.^12^^,^^18^, ^19^, ^20^, ^21^ These findings complement existing research by incorporating a randomized approach to include the general ED population, rather than only those presenting with psychiatric crises. We also describe a high prevalence of mental health symptoms, particularly among individuals with socioeconomic barriers such as lack of employment highlighting the intersecting needs of mental health and SDOH among the ED patient population.

Our observed associations between symptom burden and demographic factors including higher anxiety and PTSD symptoms among females and increased depression among younger adults, align with previous literature.^22^^,^^23^ Moreover, individuals with symptoms of mental health conditions often confront intricate social challenges that affect their access to care. We found that over 45% of our sample reported income below $35,000, 58% were unemployed, and 15.4% lacked high school completion. The presence of these SDOH is linked to heightened obstacles in accessing appropriate treatment.^24^

The typical visit to the ED is often interspersed with periods of idle time during which patients are waiting for testing to be completed, assessments by various healthcare providers, and even waiting for bed placement resulting in periods of inactivity. Implementing feasible screening programs during these periods could potentially result in identification of elevated mental health symptoms, determine potential interventions, and provide treatment resources. Evidence-based approaches such as SBIRT (Screening, Brief Intervention, and Referral to Treatment) have shown promise for addressing substance use disorders in EDs and could potentially be adapted for mental health conditions.^25^ However, screening is only a first step. Overcoming barriers to clinician referral to outpatient resources can be a challenge.^19^, ^20^, ^21^

In the present study, we found that a considerable number of ED patients exhibited elevated symptoms of mental health disorders. These findings highlight the ED as a crucial setting for early intervention in this population. The high prevalence of anxiety, depression, and PTSD symptoms underscores the potential to leverage natural waiting periods for ED-based mental health screening. Implementing systematic screening protocols may facilitate earlier identification and treatment, potentially mitigating symptom escalation and reducing future ED utilization.

## Author Contributions

JL, EH-D, JLB, MSL, CF, JES, JL, RSB, AKL, JAF, DB, JJB, MVP, RMA, AC, DP, KPH, and BEP contributed to the conception and design of the study, data acquisition, or analysis and interpretation of data. All authors were involved in drafting the manuscript or revising it critically for important intellectual content. All authors approved the final version to be published and agree to be accountable for all aspects of the work.

## Funding and Support

By *JACEP Open* policy, all authors are required to disclose any and all commercial, financial, and other relationships in any way related to the subject of this article as per ICMJE conflict of interest guidelines (see www.icmje.org). The Ohio Attorney General provided funding for the research and execution of the parent study to the institution for MSL, CF, JES, JL, RSB, JAF, DB, MVP, RMA, and BEP. The National Institute on Drug Abuse (Grant/Award Number: K08DA049948) supported manuscript development for BEP.

## Conflict of Interest

Brittany Punches reports financial support was provided by National Institute on Drug Abuse. Caroline Freiermuth reports financial support was provided by Office of the Ohio Attorney General. Brittany Punches has an editorial role for JACEP Open. Given her role as a decision editor, she had no involvement in the peer review of this article and had no access to information regarding its peer review. Full responsibility for the editorial process for this article was delegated to another journal article. Other authors declare that they have no known competing financial interests or personal relationships that could have appeared to influence the work reported in this paper.
